# A Hydrophilic Polyurethane Foam Containing *Nigella sativa* Oil as a Wound Dressing

**DOI:** 10.1155/bmri/6627611

**Published:** 2025-11-30

**Authors:** Sara Darakhshan, Sattar Mirzaie, Hadi Hossainpour, Mohsen Zhaleh, Seyran Kakebaraei, Reza Tahvilian

**Affiliations:** ^1^ Department of Biology, Faculty of Science, Razi University, Kermanshah, Iran, razi.ac.ir; ^2^ Pharmaceutical Sciences Research Center, Health Institute, Kermanshah University of Medical Sciences, Kermanshah, Iran, kums.ac.ir; ^3^ Peptide Chemistry Research Center, K. N. Toosi University of Technology, Tehran, Iran, kntu.ac.ir; ^4^ Behbahan Faculty of Medical Sciences, Behbahan, Iran, modares.ac.ir; ^5^ Department of Anatomical Sciences, School of Medicine, Kermanshah University of Medical Sciences, Kermanshah, Iran, kums.ac.ir; ^6^ Medical Biology Research Centre, Kermanshah University of Medical Sciences, Kermanshah, Iran, kums.ac.ir

**Keywords:** antibacterial activity, hydrophilic foam, *Nigella sativa* oil, polyurethane, wound dressing

## Abstract

**Purpose:**

Some challenges with current wound dressings include limited availability of advanced options and antimicrobial effectiveness. Polyurethane (PU) foams could be suitable choices as wound dressings once their disadvantages are resolved. PU wound dressings with antimicrobial and therapeutic properties are emerging as valuable options to prevent wound infection and improve the healing process. This study is aimed at developing a hydrophilic antimicrobial PU foam incorporated with *N. sativa* oil (NSO) for potential use as a wound dressing material.

**Methods:**

In the formulation of PU foam, polyethylene glycol (PEG) and glycerine ethoxylate were utilized to improve the hydrophilicity. PU foams were subjected to detailed analysis using electron microscopy, FTIR, mechanical properties, liquid absorption, porosity measurement, cytocompatibility, in vitro antibacterial assay, and animal evaluation.

**Results:**

FTIR confirmed the linkages of polyols and isocyanate in PU as well as the presence of NSO in the foam structure. The prepared foams had high porosity with homogeneous and interconnected pores. The addition of NSO to hydrophilic PU foam caused increases in tensile strength, elongation at break, and water absorption. The results of the storage experiment showed that NSO‐PU foam remained stable under high humidity and temperature conditions. NSO‐PU exhibited no significant toxicity on human dermal fibroblasts in the MTT assay. The presence of NSO also gave the foam antibacterial activity against *Escherichia coli* and *Staphylococcus aureus*. Histological study showed enhanced wound healing capability of NSO‐PU. In NSO‐PU, a thin epidermis composed of keratinocytes was observed at the wound site and the collagen deposited around the wounds treated with NSO‐PU was organized and close to normal skin tissue.

**Conclusion:**

These results indicate that this material can be used as a hydrophilic antibacterial wound dressing.

## 1. Introduction

Wound exudate can significantly hinder the skin wound healing process by creating an environment for bacteria and fungi to thrive. To prevent wound infection, two well‐known strategies include using wound dressings with the ability to absorb exudate [1] and incorporating antibacterial agents into the dressings [2]. Polyurethane (PU) foam is one of the most widely used wound dressings [3]. PU foams possess properties that enable the permeability of oxygen and water, allow the absorption of exudates, prevent microbial invasion, have changeable mechanical properties, and provide comfort at the wound site [4]. In addition, these dressings are more economical than natural polymer‐based materials (e.g., collagen, gelatin, chitosan, and fibrin). Although PU‐related dressings have been commercialized, there are still many functional deficiencies such as healing capability, antimicrobial properties, and hydrophilicity [2, 5]. These shortcomings have led to increased treatment costs, less controllable wound infections, longer treatment times, and decreased patient comfort [6]. To tackle these, several studies have modified PU by incorporating some agents [5]. Over the last decade, various active compounds have been added to PU to achieve functional wound dressings. Among these, antimicrobial compounds [7, 8], nanoparticles [9, 10], growth factors [11], herbal active components [12, 13], and hydrophilic polymers [14] have been utilized.

PU foams are considered immobilization materials for adsorbents due to their high surface area and open porous structure [12]. Ingredients such as chitosan, carboxymethyl cellulose, and sodium polyacrylate (SPA) have been included in PU foam to improve absorption capability [15, 16]. Additionally, the hydrophilicity of PU can be adjusted by controlling its chemical structure, the molecular weight of polyol, the content of hard and soft segments, and the synthesis method [17]. Hydrophilic PU is prepared by introducing hydrophilic polymers into the soft segment of the PU backbone. Polyethylene glycol (PEG) is one of the most commonly used hydrophilic polymers [11, 18]. Hydrophilic PUs offer several benefits including softness, air permeability, maintenance of a moist environment and high absorbency for exudates [1, 19–21].

*Nigella sativa* is one of the most extensively studied medicinal plants, with its seeds having numerous applications in pharmacology [22]. *N. sativa* seeds contain various chemical components including fixed and essential (volatile) oils, proteins, amino acids, carbohydrates, alkaloids, saponins, crude fibers, vitamins, and minerals [23]. *N. sativa* oil (NSO) and the most biologically active compound of the oil, thymoquinone (TQ), have shown beneficial wound‐healing properties including immunomodulatory, antimicrobial, and antioxidant effects [22, 24].

In this study, our aim was to fabricate a hydrophilic PU foam by incorporating PEG and glycerin ethoxylate (GE) into the formulation, along with NSO in the foam structure. Previously, we developed a hemostatic PU sponge with tannic acid, tranexamic acid, and kaolin as coagulant agents [25]. The main issue we addressed in this research was the nonhydrophilic nature of the PU. In the current study, a hydrophilic PU wound dressing was fabricated comprising NSO as an antibacterial agent. This PU foam was thoroughly analyzed for its morphological, structural, mechanical, antibacterial, and biological properties.

## 2. Materials and Methods

### 2.1. Preparation of Foam

PPG (Mw 2000), PEG (Mw 1000), and GE (Mw 700) were kindly provided by Isfahan Copolymer Co. (Isfahan, Iran). Silicone copolymer surfactant, amine catalyst (Dabco 33*-*LV), tin catalyst (KOSMOS T9), and toluene diisocyanate (TDI) 80/20 were purchased from Sharif Urethane Polymer Company (Tehran, Iran). The PU foams were fabricated following the method described in our previous work [25]. The isocyanate index for the synthesized foams was 91. The PPG‐to‐PEG ratio was 9:1, and GE was added at 5% w/w relative to polyol. PPG, PEG, GE, silicone copolymer surfactant, catalysts, and deionized water were combined to form a viscous translucent liquid before being mixed with TDI. After the addition of TDI, the mixture was stirred continuously for 15–20 s using a mechanical stirrer. It was then poured into small individual wooden boxes measuring 12 × 12 × 5 cm, and the lids were closed to allow foam formation in the enclosed space. The mixture was left for 24 h at room temperature until foam formation was complete. PEG 1000, which is in a solid state, needed to be heated to 37°C–40°C; once melted, it was promptly added to the reaction mixture and thoroughly mixed. The reaction was conducted at room temperature with 20%–30% humidity. *N. sativa* seeds were purchased from the market and their oil was extracted using the cold press method at a temperature below 45°C. For the NSO‐PU foam, NSO was added to polyols and mixed well using a mechanical stirrer for 5 min, after which the other ingredients were added to the mixture. The amount of NSO used was 10% of the polyols. The commercial PU foam Askina Foam was used as the positive control group. Askina is an absorbent nonadherent foam dressing designed to absorb and retain moderate wound exudate while providing a moist environment to promote healing. This foam does not contain any active ingredients.

### 2.2. Characterization of Foams

#### 2.2.1. Fourier Transform Infrared (FTIR)

The FTIR spectra of the PU foams were recorded in attenuated total reflectance (ATR) mode on the FTIR spectrometer (Shimadzu IRPrestige‐21, Kyoto, Japan). Samples were cut into cubes of 0.5 × 0.5 × 0.5 cm^3^ and placed on the stage. The spectra were recorded at room temperature within the wavenumber range of 4000–400 cm^−1^, with an average of 32 scans.

#### 2.2.2. Scanning Electron Microscopy

The PU foams were cut, mounted onto aluminum stubs, and sputter‐coated with gold. Morphological studies were conducted using a scanning electron microscope (Hitachi, SU3500) at 15 kV. The pore size of the PU foams was measured from the images (randomly 100 cells in each sample) using ImageJ.

#### 2.2.3. Density

The length, width, and thickness of the cubic specimen 10 × 10 × 10 mm^3^ were measured using a vernier caliper (Mitutoyo Digimatic Caliper 500, Tokyo, Japan). The samples were then weighed in grams, and their density was calculated and reported in grams per cubic centimeter. The density values for five specimens were averaged for each sample.

#### 2.2.4. Liquid Absorption

Absorption of foams was measured by weighing samples after they were placed in deionized water or phosphate‐buffered saline (PBS) (pH 7.2) at room temperature. Dry samples were cut to 1 × 1 cm and their weights were recorded as Wd. The swollen samples were then removed from the liquid, and excess moisture was wiped off their surfaces using paper tissue. The weights of the swollen samples were recorded as Ww after 1, 3, and 6 h. Each sample was tested five times. The swelling ratio was calculated using Equation (1) as follows:

(1) Swelling ratio=Ww−WdWd×100.

#### 2.2.5. Mechanical Properties

The tensile strength of PU foams was measured using the STM‐1 testing instrument (Santam Eng. Design Co., Iran) in accordance with ASTM standard D3574. The samples, with dimensions of 30 mm in length, 20 mm in width, and 10 mm in thickness, were fixed in a designed probe and mechanical analysis was conducted in the longitudinal direction on both sides at a stretching speed of 2 mm/min. The experiments were carried out at room temperature with a humidity level between 20% and 30%. Three samples were tested for each group.

#### 2.2.6. Stability Test

PU foams were sterilized by UV irradiation for 1 h on each side. Afterward, they were placed in aluminum foil pouches and sealed to protect them from air and humidity. They were then stored at 40^°^C ± 2^°^C and 75*%* ± 5*%* relative humidity for 3 months. Following this period, the weight and absorption capacity were measured and compared with their respective initial values. Three samples were tested in each trial.

#### 2.2.7. Antibacterial Activity

*Staphylococcus aureus* (ATCC 25923) and *Escherichia coli* (ATCC 25922) were the chosen bacteria for the experiment. Mueller–Hinton agar served as the bacterial growth medium. The agar surface was inoculated with a swab dipped in a cell suspension of a 0.5 McFarland turbidity standard, approximately equivalent to 1 ± 10^8^ colony forming units per milliliter (CFU/mL). PU dressings were punched out as circles and sterilized with a 30‐min UV treatment. The disc diffusion method was used to evaluate the antimicrobial properties of the PUs. The plate was divided into three sections: a positive control (tetracycline disk, 30 *μ*g), raw PU, and NSO‐PU samples. These samples were placed on the plates and then incubated for 24 h at 37°C. After the incubation period, images of the samples were taken, and the diameters of the inhibition zones were measured.

#### 2.2.8. Cytotoxicity

To assess the effect of compounds used in foam production on cell viability, PU foams were sterilized using UV irradiation for 30 min on each side. They were then placed in DMEM for 24 h to allow their compounds to transfer into the media (100 mg of foam in 1 mL of medium). Human dermal fibroblasts (HDFs) were cultured in DMEM supplemented with 10% fetal bovine serum (FBS) and 1% penicillin/streptomycin. The cells were incubated in a humidified incubator at 37°C with 5% CO_2_. A total of 3 × 10^4^ cells were seeded into each well of a 24‐well plate. After 24 h, 1 mL of medium containing materials leached from each foam was added to each well (supplemented with 2% FBS). Cell viability was evaluated 48 h after treatment using the MTT assay. After removing the culture medium from the wells, 500 *μ*L of medium containing 0.5 mg/mL MTT solution (Sigma‐Aldrich, United States; in PBS) was added to each well. The plate was then incubated for 4 h at 37°C. Following the incubation, the medium was removed, and 500 *μ*L dimethyl sulfoxide (DMSO) was added to each well. The plate was shaken for 10 min. The optical density was measured using a microplate reader (Synergy H1, BioTek Instruments, United States) at 490 nm. One group was treated with culture medium only as a negative control, while the other groups were compared with this control group. The test was conducted in triplicate.

#### 2.2.9. Wound Healing Assessment

The animal experiments were conducted in accordance with guidelines approved by the Research Ethics Committees of Kermanshah University of Medical Sciences (Approval No: IR.KUMS.AEC.1402.049). Eighteen male Sprague–Dawley rats (160–200 g) were randomly divided into three groups (*n* = 6): a negative control group covered with cotton gauze only, a positive control group that received commercial PU Askina Foam, and a group that used NSO‐PU. Prior to the experiments, the animals were anesthetized with an intraperitoneal injection of ketamine and xylazine mixture. The rat’s dorsal skin was shaved using an electric shaver and a full‐thickness wound with dimensions of 2 × 2 cm was created on each animal. Dressings were wrapped with a cotton bandage to prevent them from detaching from the body. Dressings were applied precisely to the wound site for all groups and changed on Days 4, 7, and 10. Each rat was housed in a separate cage with free access to standard pellets and water. On Day 14, the rats were anesthetized with an intraperitoneal injection of a ketamine (190 mg/kg) and xylazine (4 mg/kg) mixture and skin tissues were harvested and fixed in 10% formaldehyde for 72 h. The tissue sections were embedded in paraffin, cut into 5‐to‐6‐*μ*m‐thick sections, and placed on glass microscope slides. The samples were then stained with hematoxylin and eosin (H&E) or Masson′s trichrome.

### 2.3. Statistical Analysis

The results were reported as the mean values with standard deviation (SD). Differences among the groups were evaluated using one‐way analysis of variance (ANOVA) and Student′s *t*‐test. Statistical significance was considered when *p* < 0.05. Statistical analysis was conducted using SPSS (IBM SPSS Statistics 21).

## 3. Results

PU foams were synthesized using different polyols: PPG 2000 (nonhydrophilic foam) or a mixture of PPG 2000, PEG 1000, and GE 700 (raw hydrophilic PU). The NSO‐PU resulted from the addition of NSO to hydrophilic PU. Figure 1a shows the FTIR spectra of PUs. During the reaction of the isocyanate group (−NCO) in TDI and the –OH group in polyols, the polyols exhibited a disappearance of the –OH peaks in the region of 3480 cm^−1^. Peaks related to urethane linkages were observed at 1729 cm^−1^ (carbonyl groups) and 1540 and 1261 cm^−1^ (–NH bonds). PEG has linear –CH2 bonding and a peak from –CH vibration was observed at 2967 and 2865 cm^−1^. The peak at 1600–1700 cm^−1^ corresponded to C=O stretching of urea [13, 26]. The addition of NSO only had a slight effect on the chemical structure of the foam.

Figure 1(a) FTIR spectra, (b) SEM images, (c,d) mechanical properties, and (e) uptake ability of nonhydrophilic PU, raw PU, and NSO‐PU foams.  ^∗^ *p* < 0.05,  ^∗∗^ *p* < 0.01, and  ^∗∗∗^ *p* < 0.001.

**Figure:**
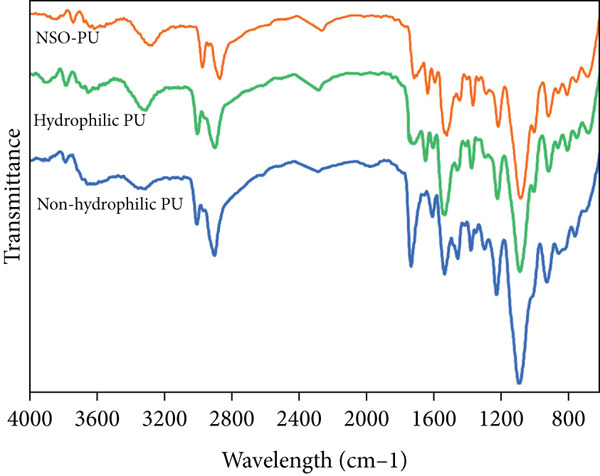
(a)

**Figure:**
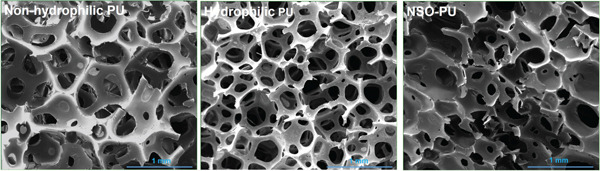
(b)

**Figure:**
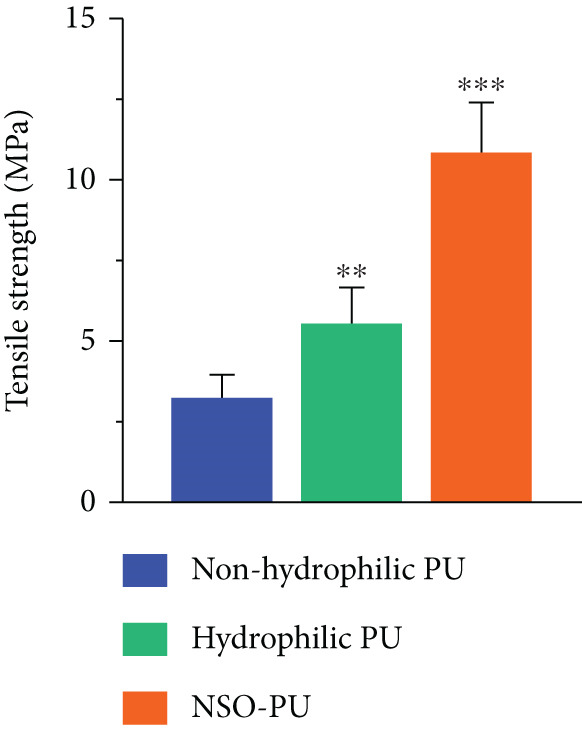
(c)

**Figure:**
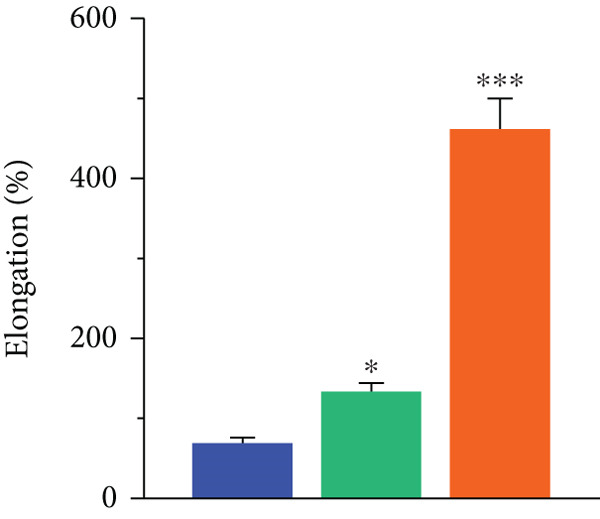
(d)

**Figure:**
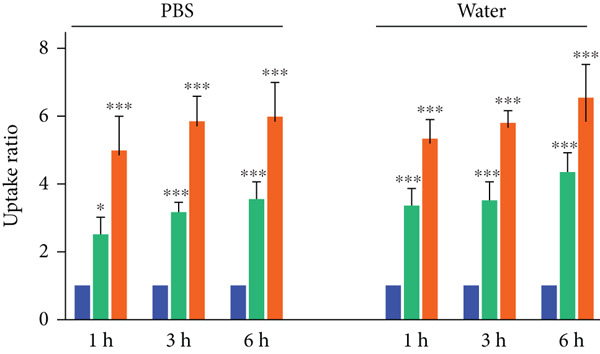
(e)

SEM images confirmed the porous structure of nonhydrophilic, raw PU, and NSO‐PU foams (Figure 1b). In all foams, the pores were interconnected, which is favorable for porous materials dedicated to wound management. All foams consisted of open cells, but the NSO‐PU foam showed a tendency towards a less homogeneous and more deformed cellular structure (Figure 1b). Modification with NSO did not significantly affect the porosity of PU foam (raw PU = 87% vs. NSO‐PU = 79%) or the pore sizes (250–400 *μ*m for raw PU vs. 300–500 *μ*m for NSO‐PU). There were no significant differences in the densities of the foams modified by NSO compared with raw PU (0.048 vs. 0.041 g/cm^3^). This result may be due to the less expansion of the polymer mass caused by the addition of oil, altering the mass/volume relationship of the system. The density of nonhydrophilic PU was 0.061 g/cm^3^, indicating that hydrophilic foam can absorb more liquids due to its lower density compared with the nonhydrophilic formulation.

To evaluate the mechanical performance of foams, we measured tensile strength and elongation at break (Figure 1c,d). The nonhydrophilic PU had relatively low levels of tensile strength (3.2 MPa) and elongation at break (72%). Tensile strength values (MPa) for raw PU and NSO‐PU foams were 5.6 and 10.7, respectively, while elongation at break (%) were 142 and 474, respectively. The results indicated that the tensile strength and elongation of raw PU and NSO‐PU were significantly higher than those of nonhydrophilic PU.

One of our objectives was to investigate the absorption capacity of the dressing, so we conducted an absorption test. An absorption test was conducted on PU foams using distilled water and PBS. The results showed that all PU foams absorbed water and PBS quickly within the first hour, then the absorption rate slowed down at 3 and 6 h. A comparison was made to nonhydrophilic PU (PPG as polyol only) as the control. After 1 h of immersion in PBS, raw PU showed a 2.6‐fold increase while NSO‐PU showed a 5.3‐fold increase compared to the control (Figure 1e). The water and PBS uptake rates of NSO‐PU after 6 h of immersion were 610% and 660%, respectively. These findings suggest that the absorption rate in the foam containing NSO was higher than in raw PU.

The results of storage experiment showed that both raw PU and NSO‐PU foams remained stable under high humidity and temperature conditions. There were no significant changes in material weight observed after 90 days of preparation for both raw PU and NSO‐PU (*p* > 0.05).

**Figure 2:**
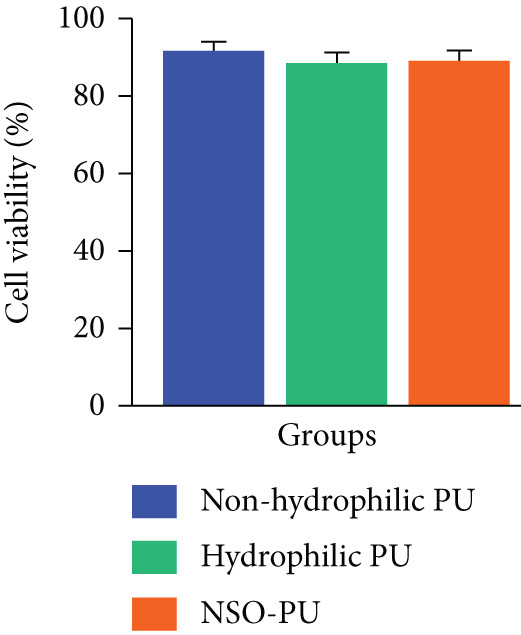
The cytotoxicity of nonhydrophilic PU, raw PU, and NSO‐PU dressings on human dermal fibroblasts (HDFs) by MTT assay. These results are the average of triplicates.

**Figure 3:**
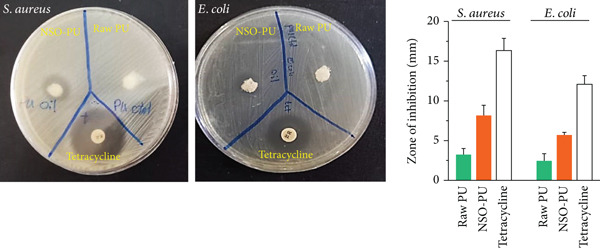
The antibacterial activity of PU foams against gram‐positive (*S. aureus*) and gram‐negative (*E. coli*) bacteria. The experiments were repeated three times.

Following treatment with foams, there was no significant difference observed in cell viability (*p* > 0.05). The viability of cells treated with nonhydrophilic PU was 91% after 48 h, while cells treated with raw PU and NSO‐PU showed a viability of 89% and 86% compared with the no‐treatment control (Figure 2). Raw PU and NSO‐PU exhibited a slight cytotoxic effect likely caused by trace residual solvents or chemical agents. The antibacterial activity of PU foams was investigated using the ZOI method (Figure 3). The inhibitory effect was assessed based on the clearance zones around the materials. The measured ZOI of raw PU was 8.1 mm for *S. aureus* and 5.7 mm for *E. coli* (Figure 3). The small amount of antibacterial effect that raw PU foam had could be due to the antibacterial properties of PEG and glycerol [27]. The inhibitory zone of NSO‐PU against *S. aureus* and *E. coli* was 15.4 and 10.3 mm, respectively (Figure 3).

**Figure 4:**
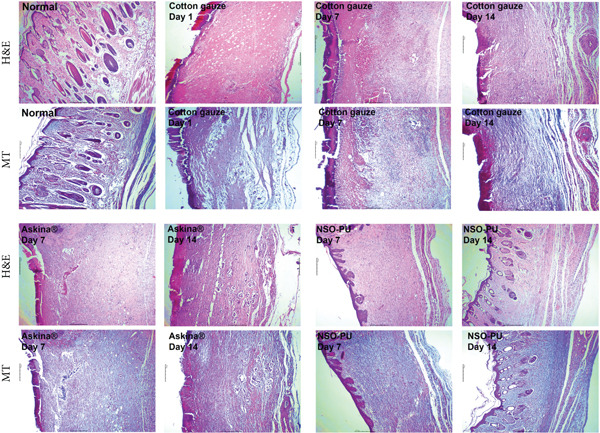
Histological assessment of wounds treated with cotton gauze, Askina, and NSO‐PU using H&E staining and Masson′s trichrome staining.

The in vivo full‐thickness excision wound model clearly demonstrated the healing effects of NSO‐PU foam (Figure 4). The effects of NSO‐PU foams on wound healing were examined through H&E staining and MT staining. In intact skin tissue, dermis components such as hair, sebaceous glands, and blood vessels have normal morphology and position. On the first day after wound induction, damage affected all layers. The creation of empty spaces due to their removal is evident and the tissue is subject to bleeding and blood coagulation. At Day 7, in the gauze‐treated group, no signs of epidermis and dermis repair can be observed. Inflammation and congestion are still noticeable and the tissue is in granulation state. In the Askina group, the tissue began to be restored in terms of morphology, and the deposition of collagen bundles was evident. Inflammation was reduced and signs of vascularization were observed. The epidermis was poorly formed and had an abnormal morphology. In the NSO‐PU group, the epidermis had a close‐to‐normal morphology but the thickness was less. The collagen deposition status was appropriate, and inflammation and congestion were markedly decreased in the dermis. On Day 14, in the gauze‐treated group, the epidermis had a stable morphology but had not yet normalized. A few sparse and scattered collagen fibers were visible. Sweat and sebaceous glands and hair follicles were not observed. The newly formed dermis in the wound region was not covered with the epithelial layer and appeared thinner than the adjacent intact tissue. In NSO‐PU, a thin epidermis composed of keratinocytes was observed although its thickness was slightly reduced. Sweat and sebaceous glands and hair follicles were formed. In both Askina and NSO‐PU, the dermis layer resumed to an almost normal state; however, the collagen deposited around the wounds treated with NSO‐PU was more organized and denser.

## 4. Discussion

Despite its many advantages, PU is considered a passive wound dressing, with its major defects being hydrophobicity and lack of functional properties. A large number of studies have focused on fabricating PU foams incorporating compounds aimed at achieving better liquid uptake and additional bioactivities [21]. The properties of PU can be adjusted by the chemical structure and molecular weight of polyols [17]. In the current study, we synthesized a hydrophilic PU foam based on a combination of PPG, PEG, and glycerol ethoxylate. It could be argued that the increased absorption ability is a result of the hydroxyl groups present in the foam, which are provided by the PEG and glycerol. This led to increased interaction between PU and liquids, resulting in the uptake of more water molecules. The absorption rate in foams containing PEG was higher compared with foam containing PPG only. An absorbent PU foam modified by PEG 400 and triethoxysilane (APTES) was fabricated as a wound dressing. This dressing demonstrated a shortened inflammatory phase and enhanced collagen deposition in a diabetic wound animal model. The uptake rate of the foam reached ~1500% in 10 min, significantly higher than that of PolyMem foam [28]. Shifting to hydrophilic polyols in foam formulation can not only improve fluid absorbency but also facilitate the release of incorporated active compounds [11]. PU foam obtained from ethylene oxide/propylene oxide random copolymer and PEG as polyols containing recombinant human hepatocyte growth factor *(*rhGF) showed an accelerated healing effect for diabetic wounds by promoting wound contraction, re‐epithelialization, and collagen deposition. This foam showed water absorption of approximately ninefold higher, with water retention fourfold higher compared with pure PU foam [11]. A study assessed the use of castor oil and glycerol as polyols for producing PU as a biomaterial, which was hydrophilic and biocompatible, with mechanical properties that could be altered by adjusting the amount of glycerol as a crosslinker for PU [14]. Our results showed that the mechanical strength of the foam increased significantly in the presence of NSO. The hydrophilicity of the foam can also be augmented by adding some compounds. In two studies, SPA, a superabsorbent, was added to PU foams, leading to a notable increase in water absorption [16, 29]. Encouraged by these studies, we decided to incorporate SPA into our hydrophilic foam (data are not shown). However, the results were unsatisfactory as some of the SPA beads were released from the foam after exposure to water, suggesting that the particles were not properly embedded in the foam.

We added NSO to the foam to provide antimicrobial properties. This antibacterial effect was observed against *S. aureus* and *E. coli*. The ameliorative effects of *N. sativa* in oil or extracts, and TQ on the wound healing process were attributed to their anti‐inflammatory, antioxidant, and antibacterial properties [30]. Studies have confirmed that NSO and TQ exhibited inhibitory effects against *E. coli*, *Streptococcus faecalis*, *S. aureus*, *Pseudomonas aeruginosa*, and *Bacillus subtilis* [31, 32]. In a recent study on periodontal pathogens, NSO inhibited the growth of *Aggregatibacter actinomycetemcomitans*, *Porphyromonas gingivalis*, *Tannerella forsythia*, and *Prevotella intermedia* [33]. TQ effectively degraded biofilms of *S. aureus*, *P. aeruginosa*, *E. coli*, and *B. subtilis* by producing reactive oxygen species [34].

Our results demonstrated that incorporating NSO in PU resulted in enhanced mechanical strength. This increase can be attributed to increasing elasticity with the plastic‐like effect of oil. In a bio‐based PU, linseed oil as polyol improved the tensile strength of films to about 50 MPa [35]. In a study, PU foams modified with 5 wt% curcumin showed increased compressive strength, higher mechanical strength, and improved stability. Moreover, the addition of curcumin exhibited antibacterial properties against *E. coli* and *S. aureus* [36]. In another study, nonisocyanate PU foams derived from lignin were created, with the addition of a silver nanoparticle solution during foaming. This lignin‐based composite foam displayed good mechanical strength and achieved a 95% bactericidal efficacy against both *E. coli* and *S. aureus* within 4 h. Evaluation in mice revealed that this composite foam effectively accelerated skin wound healing [37]. Incorporating silica nanoparticles enhanced the mechanical properties of PU by improving flexibility and maintaining good water absorption characteristics. Wounds treated with this foam showed faster closure rates as well as accelerated collagen and elastin regeneration in the newly formed dermis [38]. A hydrophilic PU foam containing silver‐hydroxyapatite exhibited wound healing activity by promoting re‐epithelialization and collagen deposition in an infected excision wound rat model [10]. Our results showed that PU foam containing NSO could lead to the regeneration of skin tissue at the wound site in an animal model. The epidermal layer and collagen fibers in the dermis were restored, and inflammation was significantly reduced.

In this study, a highly porous hydrophilic PU foam was prepared by incorporating glycerol ethoxylate and PEG. Additionally, antimicrobial and skin wound healing properties were introduced to the foam by including NSO. The incorporation of NSO along with the presence of PEG 1000 and glycerol ethoxylate had a positive impact on the mechanical strength and water uptake of the PU foam. This study suggests that adding other oils to PU foam could be a viable method for enhancing its properties.

## Conflicts of Interest

The authors declare no conflicts of interest.

## Funding

No funding was received for this manuscript.

## Data Availability

Data sharing is not applicable to this article, as no datasets were generated or analyzed during the current study.
